# Genetic screening strategy for children with hereditary spherocytosis in Jiangxi Province of China

**DOI:** 10.3389/fped.2024.1487121

**Published:** 2025-01-17

**Authors:** Chongjun Wu, Zhongjin Xu, Qian Wan, Feng Chen, Yao Ye, Hong Wang

**Affiliations:** ^1^ Department of Hematology, Jiangxi Provincial Children’s Hospital, Nanchang, China; ^2^ Department of Hematology, The Affiliated Children’s Hospital of Nanchang Medical College, Nanchang, China

**Keywords:** genetic screening strategy, children, hereditary spherocytosis, Jiangxi province, China

## Abstract

**Objective:**

This study aims to provide a comprehensive summary of the clinical phenotypic characteristics of children with anemia of unknown etiology, particularly focusing on the early detection of hereditary spherocytosis (HS) and exploring genetic screening strategies for this condition in childhood.

**Methods:**

The study included children with anemia whose underlying cause could not be definitively identified through routine clinical diagnosis. Clinical data was collected and genetic diagnosis of HS was confirmed using next-generation sequencing. Statistical analysis was conducted to evaluate the clinical characteristics of children with HS.

**Results:**

A total of thirty children with unexplained anemia were included in the study, resulting in a gene detection diagnostic rate of 80%. This included the identification of five non-HS-related congenital anemia genes (16.66%, 5/30) and nineteen cases of hereditary spherocytosis (HS). Upon initial diagnosis, the clinical features of HS were not significantly distinct compared to other forms of anemia.

**Conclusion:**

In Jiangxi, China, our strategy of genetic screening for these children is feasible after excluding the common causes of anemia, such as nutritional anemia, G-6-PD deficiency, thalassemia, autoimmune hemolytic anemia, and myelopoietic abnormalities in children. This is an exploration to establish a genetic screening strategy for children with HS, and more detailed genetic screening strategies need to be further studied and explored. Next-generation sequencing remains the main method for the diagnosis and differential diagnosis of HS.

## 1 Introduction

Hereditary Spherocytosis (HS) constitutes one of the most prevalent causes of Congenital Hemolytic Anemia (CHA) (1). HS can be identified across all races and can manifest at any age. The prevalence of HS within the Chinese population has been reported to be approximately 1:100,000 (2). The majority of studies have indicated that HS emerges as a consequence of protein defects resulting from disease-causing variants in genes encoding erythrocyte membrane proteins (e.g., *ANK1*, *SPTB*, *SPTA1*, *SLC4A1*, and *EPB42*), which are respectively associated with HS types 1–5 (3). Approximately 75% of HS cases exhibit an autosomal dominant pattern of inheritance (4) with an augmented incidence of *de novo* variants (5).

The complexity of genotype-phenotype correlations in HS has been a subject of interest. Early studies hypothesized that the biochemical and genetic heterogeneity of spherocytosis could represent the basis for clinical heterogeneity. With the growing list of HS disease-causing variants, attempts have been made to unravel the relationship between genotype and phenotype (6). However, a clear genotype-phenotype correlation in HS has not been consistently observed, with a broad variability in phenotypic presentation among disease-causing variants in different HS genes.

Patients with a family history of HS who present typical clinical manifestations (such as jaundice, anemia, splenomegaly) and laboratory findings (spherocytosis, elevated MCHC, reticulocytosis) can be diagnosed (7). In clinical practice, it is frequently encountered that certain cases lack a clear family history, their clinical manifestations are atypical [some cases only present acute hemolysis symptoms when infected (8)], and there is no single laboratory diagnostic index, thereby complicating the diagnosis. Many patients are misdiagnosed and missed diagnoses occur (9–11), particularly in pediatrics, which significantly impacts the quality of life of children. In recent years, with the application of next-generation sequencing technology, the number of HS cases has escalated. Although next-generation sequencing technology can precisely diagnose, comprehensively evaluate, and prevent missed or misdiagnosed diagnoses, the test is costly and its application is limited. How to select the appropriate cases for genetic testing to achieve the goal of early diagnosis is also an issue that we should consider.

## 2 Methods

### 2.1 Source of cases

The cases originated from children with anemia whose cause remained undetermined through routine clinical diagnosis in the Hematology Department of our hospital from January 2018 to July 2021. With the informed consent of the children and their guardians, 4 ml of the children's venous blood (EDTA anticoagulation) and 2 ml of the children's parents' venous blood were collected, and whole exon gene sequencing and Sanger sequencing were performed for verification.

### 2.2 Inclusion and exclusion criteria

#### 2.2.1 Inclusion criteria

The following two conditions must be concurrently met: (1) For all children with anemia, fundamental laboratory tests such as ferritin, iron determination, transferrin, total iron binding capacity, folic acid + Vitamin B12 determination, glucose-6-phosphate dehydrogenase (G-6-PD) deficiency, hemoglobin electrophoresis, Coombs test, thalassemia gene detection, and bone marrow examination were conducted to rule out nutritional anemia, G-6-PD deficiency, thalassemia, autoimmune hemolytic anemia, abnormal bone marrow hematopoiesis, and other common causes of anemia. (2) The children and their guardians consented to undergo peripheral blood genetic testing.

#### 2.2.2 Exclusion criteria

Any of the following conditions was fulfilled: (1) Patients with significant blood cell abnormalities other than anemia, such as thrombocytopenia and leukocyte abnormalities. (2) Patients with clinically explicit causes of other anemia. (3) Children and their guardians declined genetic testing.

### 2.3 Clinical data

Clinical data encompassing age, sex, blood counts, and other clinical indicators were gathered from all enrolled children and children with autoimmune haemolytic anaemia (AIHA) and thalassaemia identified through routine clinical diagnosis in our hospital. The clinical disparities between the HS group and the other groups were statistically analyzed. All indicators were obtained prior to splenectomy and blood transfusion.

### 2.4 Statistical methods

Based on the diverse types of diseases, the groups were categorized, and the indices such as Hb, RBC, MCV, MCH, MCHC, HCT, TBIL, DBIL, and IBIL were respectively counted. The statistical approach was the Mann Whitney test analysis for intergroup comparison, and a two-sided *p* < 0.05 was regarded as significantly different.

## 3 Results

### 3.1 Basic information of the research object

Thirty children whose cause of anemia could not be determined through routine clinical diagnosis in our hematology department (Table 1, Supplementary Material S1), 15 children with AIHA (Table 2, Supplementary Material S2), and 21 children with thalassaemia (Table 3, Supplementary Material S3). All the patients were from Jiangxi region of China, were not related by blood, and had varying degrees of anemia at admission.

**Table 1:** Clinical characteristics of 30 children.

Clinical characteristics	Results	Range	Reference range
Male, *n* (%)	12 (40.00%)	–	–
Age,median	3 years and 9 months	–	–
RBC (×10^12^/L)	2.52 ± 0.71	1.51–4.58	3.1–4.5
Hb (g/dl)	69.4 ± 17.41	33–109	110–149
MCV (fL)	84.40 ± 10.83	53.1–111.3	80–98
MCH (pg)	28.43 ± 4.51	14.4–42.5	25–35
MCHC (g/L)	331.70 ± 21.59	272–382	300–360
HCT (%)	20.88 ± 4.89	10.6–31.8	35–45
TBIL (μmol/L)	57.61 ± 45.58	5.8–201.3	2–22
DBIL (μmol/L)	12.04 ± 5.59	1.9–28.8	1–8
IBIL (μmol/L)	45.57 ± 44.47	3.0–184.6	1–19

**Table 2:** Clinical characteristics of 15 children diagnosed with AIHA.

Clinical characteristics	Results	Range	Reference range
Male, *n* (%)	8 (53.33%)	–	–
Age, median	2 years and 10 months	–	–
RBC (×10^12^/L)	1.81 ± 0.73	0.99–3.06	3.1–4.5
Hb (g/dl)	52.53 ± 18.72	24–89	110–149
MCV (fL)	100.59 ± 17.27	79.5–136.1	80–98
MCH (pg)	32.79 ± 4.83	27.8–44.0	25–35
MCHC (g/L)	325.72 ± 29.01	264–361	300–360
HCT (%)	17.59 ± 7.74	7.3–36.1	35–45
TBIL (μmol/L)	50.36 ± 44.08	10–196.5	2–22
DBIL (μmol/L)	11.45 ± 3.77	3.4–17.2	1–8
IBIL (μmol/L)	38.91 ± 41.90	6.6–180.9	1–19

**Table 3:** Clinical characteristics of 21 children disnosed with thalassaemia.

Clinical characteristics	Results	Range	Reference range
Male, *n* (%)	13 (61.90%)		–
Age, median	5 years and 11 months		–
RBC (×10^12^/L)	2.76 ± 0.67	1.44–4.93	3.1–4.5
Hb (g/dl)	69.76 ± 14.03	40–94	110–149
MCV (fL)	78.53 ± 6.80	56.7–89.8	80–98
MCH (pg)	25.57 ± 3.01	17.6–30.0	25–35
MCHC (g/L)	325.14 ± 19.16	278–355	300–360
HCT (%)	21.50 ± 4.34	11.7–28.0	35–45
TBIL (μmol/L)	34.49 ± 19.49	9.29–74.4	2–22
DBIL (μmol/L)	8.75 ± 3.15	4.4–14.78	1–8
IBIL (μmol/L)	25.74 ± 17.71	4.29–62.6	1–19

### 3.2 Results of genetic testing of 30 children enrolled with unexplained anaemia

Among the 30 children whose cause of anemia could not be determined through routine clinical diagnosis in our hematology department (Table 4), the overall diagnostic rate of gene detection was 80% (24/30), including 5 non-HS-related congenital anemia genes (*GPI*, *PKLR*, *GATA1*, *CUBN*, *HBB*) (16.66%, 5/30), and 19 cases were identified as HS-related genes (63.33%, 19/30). Among the 19 HS cases, 12 (63.15%) were caused by *ANK1* variants, and 7 (36.84%) were caused by *SPTB* variants. Disease-causing variants were found to be heterozygous in 19 children diagnosed with HS, of whom 15 were classified as pathogenic and 4 as likely pathogenic. The *HBB* genes was heterozygous, whereas *CUBN* was identified as homozygous. In addition, *PKLR* and *GPI* presented as compound heterozygous variants, whereas *GATA1* was found to be hemizygous. In particular, Patient 1 exhibited a heterozygous deletion of *CFHR3/CFHR1*. To maintain objectivity, we provisionally categorized this case as negative.

**Table 4:** Results of genetic testing of 30 children.

ID/References	Gene	Chromosomal location	Coding	Protein	Pathogenicity of variants
1/Present study	Negative				
2/Present study	Negative				
3/Present study	Negative				
4 (12)	*ANK1*	chr8:41580696	c.955C>T	p.R319*	Pathogenic
5 (12)	*SPTB*	chr14:65258462	c.2779C>T	p.Q927*	Pathogenic
6/Present study	*HBB*	chr11:5247907	c.215T>C	p.F72S	Pathogenic
7 (13)	*CUBN*	chr10:17127755	c.1951C>T	p.R651*	Pathogenic
8 (12)	*ANK1*	chr8:41546059	c.4276C>T	p.R1426*	Pathogenic
9 (12)	*SPTB*	chr14:65253803	c.2880C>A	p.C960*	Likely pathogenic
10 (14)	*GATA1*	chrX:48652491–48652492	c.1162_1163del	p.L388Lfs*61	Likely pathogenic
11/Present study	*SPTB*	chr14:65239969	c.5147del	p.P1716Rfs*10	Pathogenic
12 (12)	*SPTB*	chr14:65253555	c.3128G>A	p.W1043*	Pathogenic
13/Present study	negative				
14 (12)	*ANK1*	chr8:41571714–41571715	c.1858_1859del	p.L620Afs*33	Pathogenic
15/Present study	*ANK1*	chr8:41552800	c.3133del	p.R1045Afs*6	Likely pathogenic
16/Present study	*PKLR*	chr1:155269926	c.246del	p.V83Wfs*25	Pathogenic
	*PKLR*	chr1:155264136	c.1006G>A	p.A336T	Likely pathogenic
17 (12)	*ANK1*	chr8:41615556	c.226C>T	p.Q76*	Pathogenic
18/Present study	*GPI*	chr19:34887252	c.1142G>A	p.R381H	Likely pathogenic
	*GPI*	chr19:34859515	c.427C>T	p.R143W	Likely pathogenic
19 (12)	*ANK1*	chr8:41573376	c.1504–9G>A	/	Likely pathogenic
20 (12)	*ANK1*	chr8:41546059	c.4276C>T	p.R1426*	Pathogenic
21 (12)	*ANK1*	chr8:41559664	c.2395-2A>G	/	Pathogenic
22 (12)	*SPTB*	chr14:65260556	c.1825C>T	p.Q609*	Pathogenic
23 (12)	*ANK1*	chr8:41547849	c.4123C>T	p.R1375*	Pathogenic
24/Present study	Negative				
25/Present study	Negative				
26 (12)	*ANK1*	chr8:41545696–41545697	c.4358_4359del	p.E1453Afs*46	Pathogenic
27 (12)	*SPTB*	chr14:65241215	c.4873C>T	p.R1625*	Pathogenic
28 (12)	*ANK1*	chr8:41559136–41559139	c.2489_2492del	p.L830Sfs*7	Pathogenic
29 (15)	*SPTB*	chr14:65236307	c.5937+1G>A	/	Pathogenic
30/Present study	*ANK1*	chr8:41525880	c.5422G>T	p.E1808*	Likely pathogenic

* Which represent stop codons.

### 3.3 Comparison of clinical characteristics of HS and other congenital anemia

**Figure 1:**
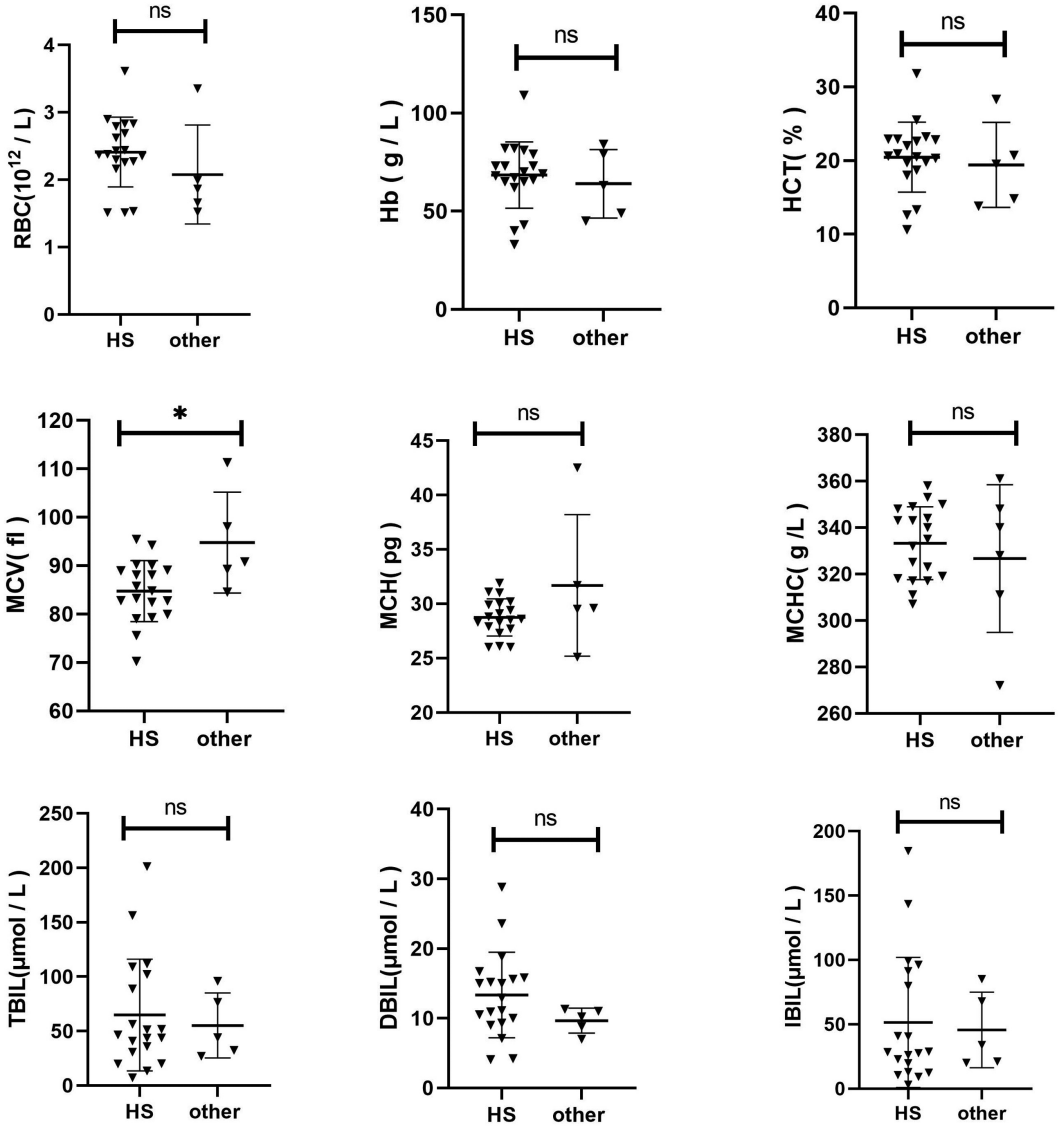
Comparison of clinical characteristics of HS and other congenital Anemia.

The Hb, RBC, MCV, MCH, MCHC, HCT, TBIL, DBIL, and IBIL were compared between the two groups (Figure 1). The MCV of HS children was lower than that of the control group (*p* = 0.0115). No significant differences were found in other indices between the two groups (*p* > 0.05).

### 3.4 Comparison of clinical characteristics between HS and gene-negative anemia of unknown origin

**Figure 2:**
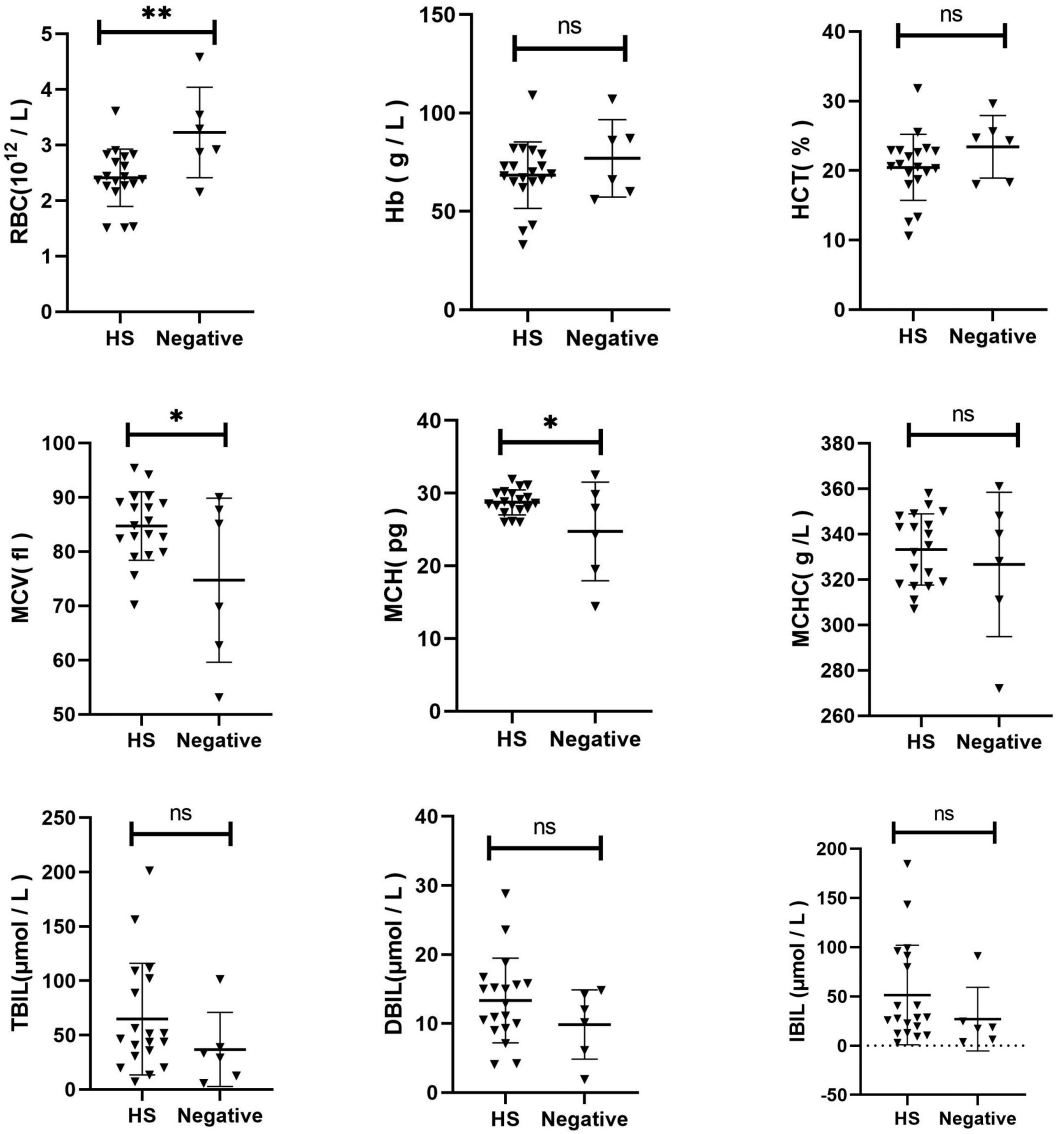
Comparison of clinical characteristics between HS and gene-negative Anemia of unknown origin.

The Hb, RBC, MCV, MCH, MCHC, HCT, TBIL, DBIL, and IBIL were compared between the two groups (Figure 2). The RBC of HS children was lower than that of the control group (*p* = 0.0076). The MCV (*p* = 0.0266) and MCH (*p* = 0.0228) of HS were slightly elevated than the control group. No significant difference was observed in other indices between the two groups (*p* > 0.05).

### 3.5 Comparison of clinical characteristics between HS and AIHA

**Figure 3:**
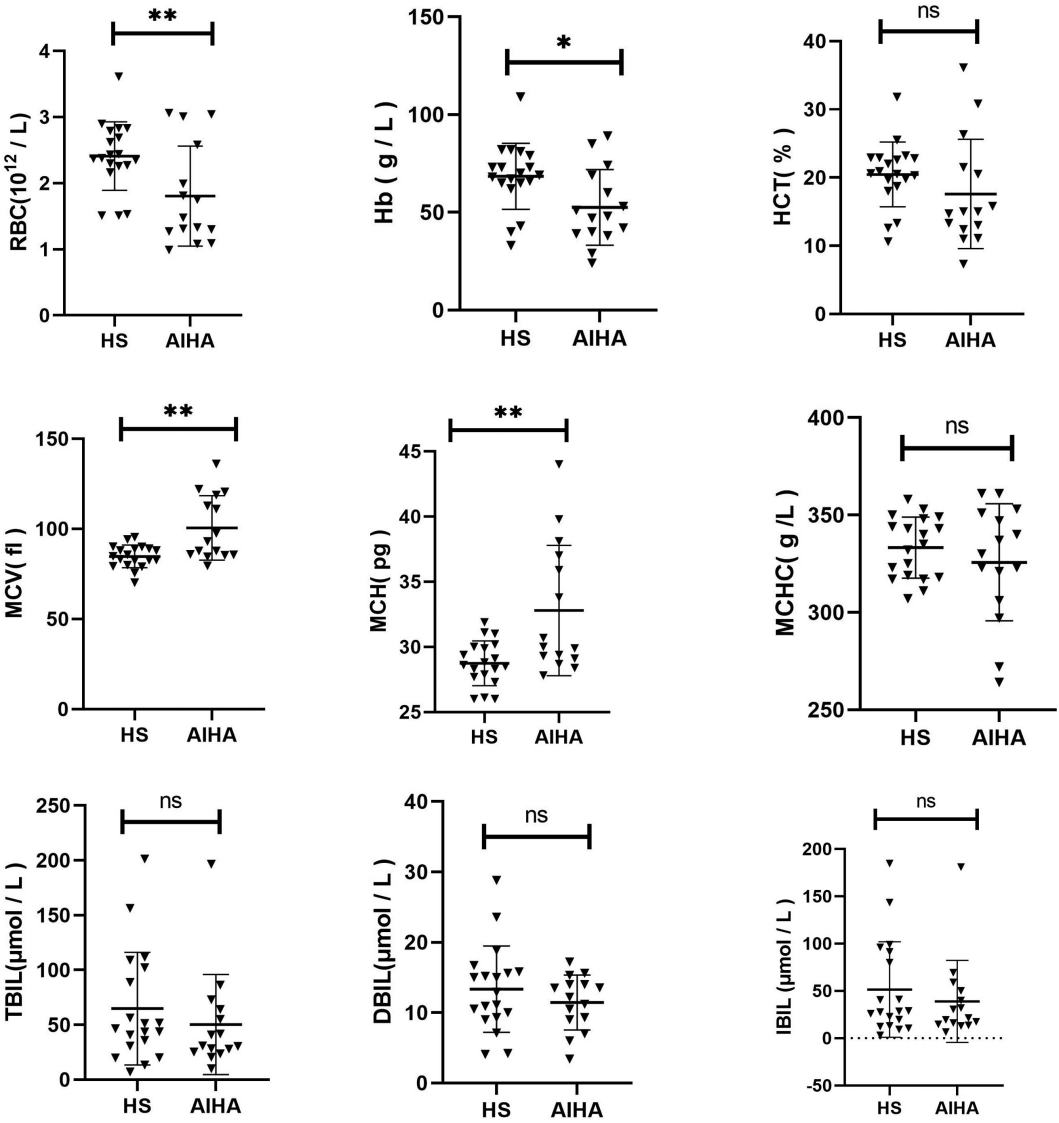
Comparison of clinical characteristics between HS and AIHA.

The Hb, RBC, MCV, MCH, MCHC, HCT, TBIL, DBIL, and IBIL were compared between the two groups (Figure 3). It was discovered that RBC (*p* = 0.0095) and Hb (*p* = 0.0157) of HS children were significantly higher than those of AIHA children. The MCV (*p* = 0.0010) and MCH (*p* = 0.0023) of HS were significantly lower than those of AIHA. No significant difference was noted in other indices between the two groups (*p* > 0.05).

### 3.6 Comparison of clinical characteristics between HS and thalassemia

**Figure 4:**
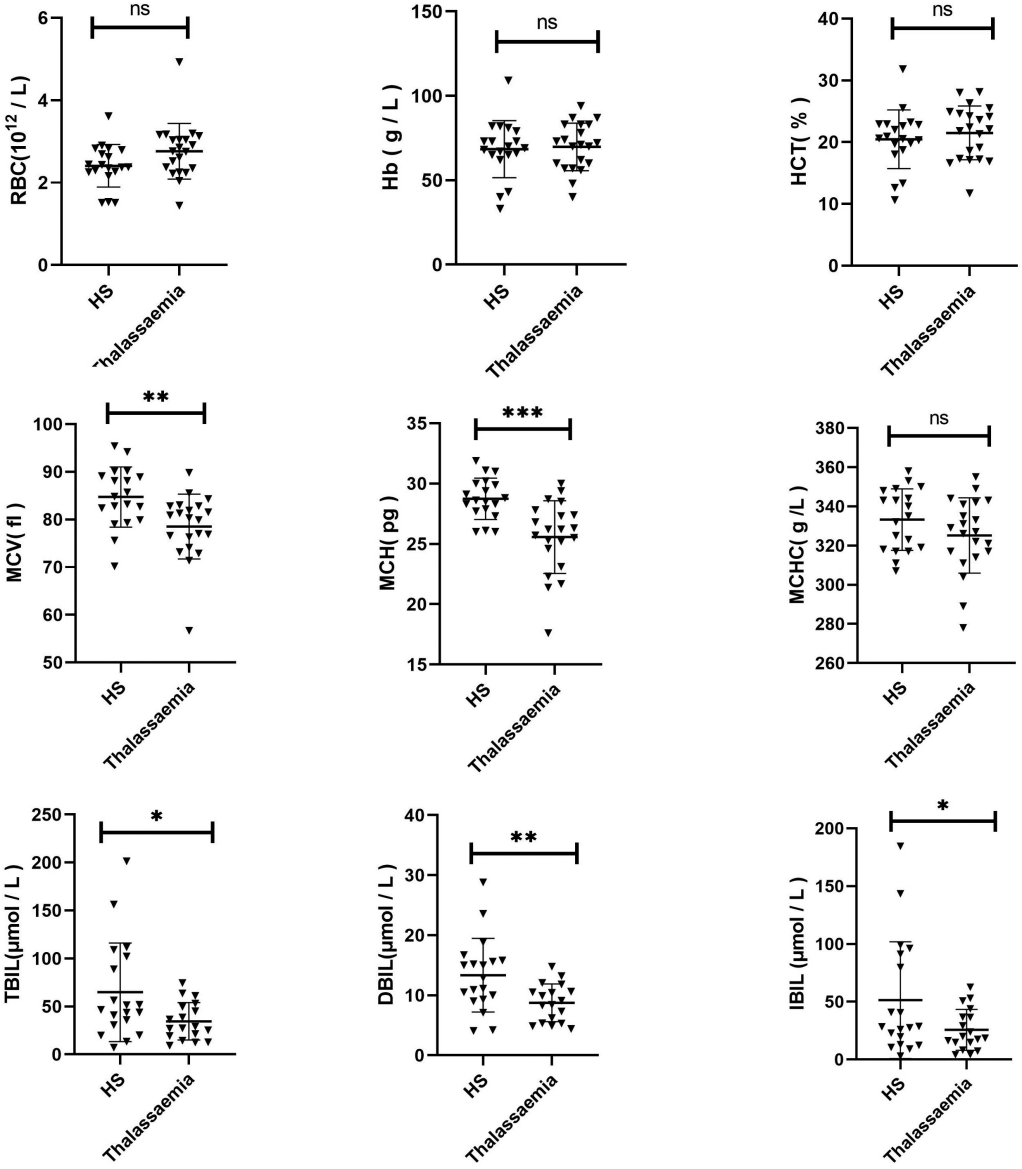
Comparison of clinical characteristics between HS and thalassemia.

The Hb, RBC, MCV, MCH, MCHC, HCT, TBIL, DBIL, and IBIL were compared between the two groups (Figure 4). It was found that MCV (*p* = 0.0051), MCH (*p* = 0.0002), TBIL (*p* = 0.0245), DBIL (*p* = 0.0073), and IBIL (*p* = 0.0488) of HS children were significantly higher than those of thalassemia children. No significant difference was detected in other indices between the two groups (*p* > 0.05).

## 4 Discussion

The World Health Organization estimates that approximately 25% of the global population is anaemic, with nearly half of this number comprising preschool children. In cases where a child with anaemia is admitted, detailed medical information, especially in regard to diet, environmental exposures, and family history, can often provide crucial insights for diagnosis. Abnormalities observed during a physical examination may indicate the underlying cause of anaemia (16). Nevertheless, in rare cases of congenital anaemia, some children remain undiagnosed despite exhaustive testing. The objective of this study was to clarify the diagnosis of anaemia of unknown origin by next-generation sequencing and to explore the feasibility of HS genetic screening strategy for children with anaemia of unknown cause in clinical routine diagnosis.

The diagnostic rates of congenital anaemia (such as hereditary spherocytosis, etc.) observed in NGS methods across different countries vary considerably, with rates ranging from 63% to 90% (17–23). The diagnostic rate of congenital anaemia in this cohort was 80%, which is comparable to the findings of an Italian study that examined 155 consecutive cases of patients with clinically suspected hereditary red cell defects from 2018 to 2020. The overall diagnostic rate of genetic testing was 84% (24). In a previous Brazilian study (25), targeted sequencing analysis of NGS in 36 samples from unrelated patients with clinically suspected hereditary anaemia identified potentially pathogenic variants in 26 cases, representing 72% of the total number of samples analysed. In our present study, a significant proportion of patients have opted to decline genetic testing for a range of reasons, with some seeking further diagnosis and treatment at other medical centres. This may result in a certain degree of bias in our data, which requires further investigation.

In this group of data, hereditary haemolytic anaemia was identified as the primary aetiology in all associated cases, with the majority ultimately diagnosed with HS. This may be attributed to the routine exclusion of common haemoglobinopathy and enzymopathy, such as thalassaemia and G-6-PD deficiency, in this group of cases. In this data set, the peripheral blood globular red blood cell count was not sufficiently specific at the initial diagnosis to provide clear indications for the disease diagnosis (26). It is unfortunate that the EMA screening and other HS-related specific laboratory tests were not conducted on this dataset prior to genetic testing. This may prove to be of relevance to our actual medical situation. It is important to note that HS is not a common disease, and not all hospitals have the capacity to complete the specific laboratory tests related to HS. Consequently, some experts have put forth the proposition that the osmotic fragility (OF) test can be utilized as an affordable diagnostic instrument in resource-limited settings, particularly in the absence of spherocytosis in peripheral blood smears (27). Nevertheless, this further illustrates that NGS can serve as an alternative to time-consuming traditional HS-related laboratory tests, particularly in paediatric settings. In the majority of cases, a diagnosis is reached through a combination of an individual's medical history, basic laboratory tests, and an NGS panel containing five genes. In the majority of cases, more comprehensive functional screening is not required (28).

In the meantime, Patient 1 exhibited a heterozygous deletion of *CFHR3/CFHR1*. In our previous work, we observed that Patient 1, an older child presenting with anemia, had other potential causes clinically ruled out. Based on this observation and relevant literature (29, 30), we suspect an association between the gene deletion and the patient's condition. We acknowledge that under specific circumstances, such deletions may be linked to the disease, particularly after excluding all other possibilities. However, it is important to note that other experts argue that homozygous deletions of *CFHR3/CFHR1* are associated with the formation of anti-factor H autoantibodies, which confer an increased risk of developing autoimmune atypical hemolytic uremic syndrome (aHUS) (31). Heterozygous deletions are generally not considered risk factors for aHUS. To preserve objectivity, we provisionally categorize this case as negative and will conduct further evaluation and follow-up in subsequent work.

A comparative analysis was conducted to examine the clinical characteristics of children with HS and those with other forms of congenital anaemia. The MCV in children with HS was slightly lower than that in the control group (*p* = 0.0115). No statistically significant differences were observed in other indices between the two groups (*p* > 0.05). Additionally, a comparison was made between the clinical characteristics of children with HS and those with gene-negative anaemia of unknown origin. The RBC in the HS group was significantly lower than that in the control group (*p* = 0.0076). Additionally, compared to the control group, the MCV and MCH levels in the HS group were slightly elevated (*p* = 0.0266 and *p* = 0.0228, respectively). No statistically significant difference was observed in the remaining indicators between the two groups (*p* > 0.05). These findings indicate that a distinctive clinical phenotype of HS does not exist. In the absence of a clinical diagnosis of HS, next-generation sequencing represents the primary diagnostic and differential diagnostic method. Moreover, the HS genetic screening strategy selected for use in our region has been demonstrated to be a viable approach.

Peripheral blood spherocytosis is a typical feature of HS, but it is also a common feature of HS and some AIHA patients. The OF test is regarded as the primary diagnostic method for HS; however, its sensitivity and specificity are relatively low, and positive results are also frequently observed in AIHA patients (32). In order to gain further insight into the clinical differences between HS and AIHA at an early stage, we conducted a comparative analysis of the red blood cell parameters of 19 children with HS and 15 children with AIHA. The results demonstrated that children with HS exhibited milder anaemia and a smaller cell volume than those with AIHA. It is regrettable that no further differences could be identified, which would benefit from further investigation with a larger sample size. This is significant for differential diagnosis between the two, particularly in instances where the Coombs test is negative.

Thalassaemia is considered a significant public health issue in our region. A comprehensive diagnostic survey for thalassaemia has been established (33). Conversely, the accurate diagnosis of RBC membranopathy and enzymopathy remains a developing field, a situation that is also prevalent in Thailand (34). In the course of our study, patient ID6 exhibited an HbA2 score of 4.7 (reference range: 2.2–3.5) during a routine examination of haemoglobin, which prompted us to suspect the presence of thalassaemia. The results of the routine genetic testing for thalassaemia (PCR + Diversion Hybridization Method, 23 common disease-causing variant sites) conducted at our hospital were unremarkable, with no abnormalities identified. In Case 6, the *HBB* p.F72S variant (resulting from c.215T>C) was identified and classified as pathogenic according to an adjusted ACMG classification (PS2 + PM1 + PM2 + PP3 + PP4). This classification is based on findings from this study and in silico analysis results: SIFT deleterious (0.01), PolyPhen probably_damaging (0.995), and the CADD_PHRED score is 24.2 that indicates a harmful variant. *HBB* F72S variant was previously classified as uncertain significance of pathogenicity in the ClinVar database and, however, several thalassemia cases with F72S variant was previously reported in the literature (35, 36) and in the LOVD database (case #000602036, https://databases.lovd.nl/shared/variants/000602036#00000017), which indicates it may be a recurrent HBB disease-causing variant. Furthermore, we ran a comparative analysis of the clinical data in order to identify any potential discrepancies between the HS and thalassaemia groups. The red blood cell size of children with thalassaemia was observed to be smaller, which is consistent with the characteristics of thalassemia (37). Additionally, the jaundice observed in HS was found to be more severe, which may be attributed to the HS disease itself or alternatively, it may be the case that only thalassaemia children who required long-term blood transfusion treatment were selected.

In addition, HS is based on defects in genes encoding major red blood cell cytoskeleton and (trans)membrane proteins, including ankyrin-1 (*ANK1*), band-3 (*SLC4A1*), *α*-spectrin *(SPTA1*), *β*-spectrin (*SPTB*), and protein 4.2 (*EPB42*). We have highlighted that with the increased use of NGS techniques, the list of unique pathogenic variants underlying HS is growing rapidly, which adds to the complexity of genotype-phenotype correlations. It is worth noting that there are differences in the assessment of disease severity due to the lack of uniform criteria (4).

The present study was conducted on the basis of the exclusion of G-6-PD deficiency. Furthermore, the results indicated the presence of two cases of anaemia resulting from RBC enzymopathy (ID16, 18). At the present time, our medical institution is unable to detect the activity levels of these two enzymes. This has also posed significant challenges to our daily medical practice. Some scholars have proposed that among the three children with Pyruvate kinase deficiency (PKD), PK enzyme activity was only moderately decreased in two cases and normal in one case, which also indicates that the detection of enzyme activity is of limited diagnostic value (38). Genetic testing has become a principal method for the rapid and accurate diagnosis of genetic disorders. NGS has transformed the diagnostic framework for HS, reducing the time and cost of associated tests.

## 5 Conclusion

Next-generation sequencing remains the primary methodology for the diagnosis and differential diagnosis of HS. In Jiangxi, China, our proposed strategy of genetic screening for these children is feasible following the exclusion of the most common causes of anaemia, including nutritional anaemia, G-6-PD deficiency, thalassaemia, autoimmune haemolytic anaemia, and myelopoietic abnormalities in children. This study represents an initial exploration of the potential for developing a genetic screening strategy for children with HS. Further research is required to fully elucidate the genetic screening strategies that could be employed in this context.

## Data Availability

The original contributions presented in the study are included in the article/Supplementary Material, further inquiries can be directed to the corresponding author.
